# Stem Cell Therapy in Acute Myocardial Infarction: A Pot of Gold or Pandora's Box

**DOI:** 10.4061/2011/536758

**Published:** 2011-05-11

**Authors:** V. K. Shah, K. K. Shalia

**Affiliations:** ^1^Interventional Cardiologist, Sir H.N. Hospital and Research Centre, Raja Rammohan Roy Road, Mumbai 400 004, India; ^2^Sir H.N. Medical Research Society, Sir H.N. Hospital and Research Centre, Raja Rammohan Roy Road, Mumbai 400 004, India

## Abstract

Stem cell therapy for conditions characterized by myocyte loss in myocardial infarction and heart failure is intuitively appealing. Stem cells from various sources, including heart itself in preclinical and animal studies, have shown the potential to improve the function of ventricular muscle after ischaemic injury. The clinical experience from worldwide studies have indicated the safety profile but with modest benefits. The predominant mechanisms of transplanted cells for improving cardiac function have pointed towards paracrine effects rather than transdifferentiation into cardiomyocytes. Thus, further investigations should be encouraged towards bench side and bedside to resolve various issues for ensuring the correct type and dosing of cells, time, and method of delivery and identify correct mechanism of functional improvement. An interdisciplinary effort at the scientific, clinical, and the government front will bring successful realization of this therapy for healing the heart and may convert what seems now a Pandora's Box into a Pot of Gold.

## 1. Clinical Need

Myocardial infarction (MI) remains a major cause of morbidity and mortality. Rapid reperfusion of the occluded coronary arteries is of great importance in salvaging ischemic myocardium and limiting the size of infarct. This reduces early complications and improves survival rates. Unfortunately, myocardial necrosis starts rapidly before reperfusion can be achieved in most of the patients, leaving an infarct zone that contains nonfunctional myocytes that are remodeled into the scar tissues surrounded by region of ischemia. Contemporary reperfusion strategies using percutaneous interventions aided with pharmacotherapy and mechanical devices have shown to resolve the ischemia with only modest improvements in global Left Ventricular Function (LVF) as evidenced by 2% to 4% increase in LV Ejection Fraction (LVEF) at six months after an acute MI [1, 2]. This loss of viable myocardium initiates a process of adverse ventricular remodeling and a downward spiral leading to congestive heart failure. This is followed by repeated hospitalization and increased economic burden on the society with 50% of the patients dying within five years of the diagnosis. Scar tissue is incapable of performing the vital function of cardiac muscle and suffers from decreased cardiac output. Revival of the cardiac tissue in infarct zone can enhance the functional activity of the heart. Thus, heart muscle salvage after heart attack is the single important determinant factor for the event-free long-term survival. 

Considered as terminally differentiated organ, regenerating the myocardium was never thought of as an option for heart muscle salvage. Stem cell-based therapy became a realistic option to replace damaged heart muscles due to series of experimental findings of myocyte turnover in mammalian heart (Table 1). Evidence such as fraction of cardiomyocytes may be able to reenter the cell cycle and that limited regeneration can occur through recruitment of resident and circulating stem cells were presented [3–11]. But it was also realized that these endogenous repair mechanisms are overwhelmed by the substantial damage to the myocardium from the injury that it faces during MI. However, the existence of these endogenous repair mechanisms as well as the concept of adult stem cell plasticity suggested that cardiac repair may be achieved therapeutically in these clinical settings and gave a way for preclinical trials. Subsequent promising reports of these same trials prompted rapid initiation of human clinical trials. In the present paper, we discuss the different types of stem cells and their journey in healing the heart, certain unresolved issues and discuss key points for the design of future stem cell therapy trials.

## 2. Stem Cells

Stem cells are primitive, undifferentiated, undefined pluripotent multilineage cells that retain the ability to renew themselves through mitotic cell division and can divide and create a cell more differentiated than itself. Every single cell in the body originates from this type of cell. They are obtained not only from embryo and fetus but also from various parts of the adult body. Adult stem cells are defined as undifferentiated progenitor cells from an individual after embryonic development. Multiple tissues have been shown to contain organ-specific progenitor cells. However, adult stem cells have less potential to differentiate without assistance. Stem cells are usually classified according to the following criteria: origin, type of organ or tissue from which the cells are derived, surface markers, and final differentiation fate (Table 2). 

### 2.1. Embryonic Stem Cells (ESCs)

ESCs are totipotent stem cells derived from the inner mass of the blastocyst stage late in the first week of fertilization. They differentiate into multicellular embryoid bodies containing differentiating cells from all three germ layers, ectoderm, mesoderm, and endoderm, and are able to give rise to most somatic cell lineages [12–14]. Since the mid-eighties, it has been shown that during in vitro differentiation into cystic embryoid bodies, ESCs differentiate into beating cardiac myocytes [12] and are electromechanically coupled to the host cardiac cells [15, 16]. ESC-derived cardiac myocytes most closely resemble embryonic cardiac myocytes and express the complete repertoire of cardiac-restricted transcription factors including GATA4, Nkx2.5, MEF2C, and Irx4 [17]. In several rodent models it has been shown that, when transplanted into infarcted myocardium, ESCs-derived cardiomyocytes engraft and improve cardiac function [18–21]. However, there are certain limitations for their use. The first is the likelihood of teratoma formation at the implantation site. This can be resolved by their differentiation prior to implantation and thus yielding a pure cardiac myocyte population [22]. The second issue pertains to immunity. Once thought to be uniquely immunoprivileged, increasing evidence has demonstrated that ESCs express specific human leukocyte antigen (HLA) subclasses [23]. This raises the worry of graft rejection and might necessitate immunosuppression. However, Steroid use without concomitant stem cell implantation has been known for some time to be harmful to ischemic myocardium [24]. There is currently ongoing research to help limit the immunogenicity of the cells for allogeneic transplantation. Finally, the origin of ESCs has raised considerable ethical concerns and led to heated debates among scientists and the wider public. The recent discovery that it is possible to generate ESC-like cells, called inducible pluripotent stem (iPS) cells, by reprogramming adult somatic cells with genes regulating ESC pluripotency may resolve the ethical and immunogenic issues associated with the use of ESCs [25–27].

### 2.2. Induced Pluripotent Stem (iPS) Cells

Induced pluripotent stem (iPS) cells are the stem cells artificially derived from adult somatic cells which have been induced to express a gene profile characteristic of ESCs (Oct3/4, Sox2, KLF4, cMyc) in response to genetic engineering [25, 28–30]. iPScellsare thought to be therapeutically equivalent to ESCs, in many respects, such as the expression of certain embryonic markers (SSEA-1) and proteins, chromatin methylation patterns, doubling time, embryoid body formation, teratoma formation, viable chimera formation, potency, and differentiability. These cells are genetically identical to the donor cells [30, 31]. In contrast to ESCs, the use of iPS does not generate ethical controversies. Expansion of iPS in stem cell media can yield a sufficient number of cells that can subsequently be used for studies on cardiac differentiation. But the full extent of their potential and possible toxicity is still being assessed [28, 32].

### 2.3. Cardiac Stem Cells (CSCs)

Several groups of investigators reported that the postnatal heart includes niches of CSCs and/or cardiac progenitors with the capacity to replicate and differentiate into cardiac myocytes [18, 19, 33–39]. These cell populations included side population (SP) cells (Hoechst 33342 and Rhodamine 123 dye negative) [5, 40], cell expressing the stem cell factor c-Kit (CD117) [41], cell expressing the stem cell antigen 1 (Sca-1^+^) [42] cardiosphere-derived cells [43] and expressing the protein Islet-1 detected in the neonatal hearts. These cells are approximately 1/10th the size of adult cardiac myocytes. When isolated by repeated panning or FACS sorting, 7–10% of these cells expressed the early cardiac-restricted markers GATA4, Nkx2.5, and MEF2 [39, 41, 44]. Expression of these markers does not definitively mark a cell as cardiac in origin but does support this conclusion. Since they are cardiac in origin, perhaps such cells might provide a mechanically and electrophysiologically compatible source of cells for transplantation. These cells can be harvested from cardiac biopsies. They were demonstrated to give rise to cardiomyocytes, endothelial cells (ECs), and smooth muscle cells (SMCs) in preclinical and some animal experiments with improved LV functions. SP cells [45–47], c-kit cells [48–50], and cardiosphere cells [43, 51–54] were demonstrated to give rise to cardiomyocytes, ECs, and SMCs in preclinical and some animal experiments with improved LVF, while Sca-1^+^ CD31^−^ cells were shown to differentiate into cardiomyocytes and ECs in culture as well as in mice after MI and improved cardiac function by promoting new blood vessel formation [55]. CSCs isolated and cloned from the heart ventricles of rat subjects have been shown to be effective in the treatment of myocardial ischemia, therefore making the heart a viable source of stem cells for myocardial repair [48].

Cardiac stem cells (as well as stem cells from other tissues) appear to reside in specialized niches, which support the growth and maintenance of the stem cell pool [56, 57]. Putative niches have been localized throughout the myocardium, concentrated in deep tissue at the atria and apex [41, 58]. Recent evidence has also shown that there is a marked increase in the number and migration of such cells to the injury areas following an ischemic insult [42]. Although the different cardiac stem cell pools are small relative to the mature resident cardiomyocytes, they are believed to be the source of new cells in normal organ homeostasis as well as in stressed myocardium [59]. At present, it is unclear if the various cardiac stem cells are distinct types or whether they represent different stages of a single cell lineage. Furthermore, it appears that the cardiac stem cell pool diminishes with ageing, possibly contributing to the lack of efficacy of regeneration in elderly individuals [59].

### 2.4. Skeletal Myoblasts

Often called “Satellite Cells” which are found beneath the basal membrane of muscle fibres lie dormant till stimulated to proliferate by muscle injury or disease [60]. These cells were the first to enter the clinical arena after completion of a decade of experimental testing resulting in at least 40 studies. Myoblasts can be isolated from skeletal muscle biopsies and expanded in vitro. These studies consistently showed differentiation of implanted myoblasts into multinucleated myotubes (not cardiomyocytes) with the lack of connexin activity and absence of electromechanical coupling with the host cardiomyocytes. Despite these apparent short comings, a definite improvement in regional and global LVF was demonstrated. These data along with the clinically appealing characteristics of skeletal myoblasts (a high in vitro scalability of the initial biopsy, an advance stage of differentiation virtually eliminating tumorigenicity, and a high resistance to ischemia) paved the way for the initial human trials which started in June 2000 [61, 62]. Nonetheless, it would appear that enthusiasm for this approach is waning. However, considerations for modified or preselected products have been formulated, and a “second generation” of skeletal myoblasts modified by cell enhancement techniques have been hypothesized [63, 64].

### 2.5. Bone Marrow Stem Cells (BMSCs)

The bone marrow exemplifies a typical adult stem cell source containing different cell populations that have the potential to migrate and transdifferentiate into cells of diverse phenotypes. Unfractionated bone marrow cells contain different stem and progenitors cell populations including Haemopoietic Stem Cells (HSCs), Endothelial Progenitor Cells (EPCs), and Mesenchymal Stem Cells (MSCs). Apart from these there are *Multipotent Adult Progenitors Cells (MAPCs)* also derived from bone marrow stromal cells. They have the ability to differentiate in vitro in cells of three germ layers and differentiate into cardiac, endothelial, and smooth muscle cell phenotypes.

### 2.6. Hematopoietic Stem Cells (HSCs)

HSCs can be isolated from bone marrow cells through selective sorting for a particular set of surface antigen (Lineage negative [Lin^−^] c-kit^+^, Sca-1^+^, CD34^lo^, and CD38^hi^) [65, 66] and represent the prototypic adult stem cell population. They were shown to differentiate into cardiomyocytes in culture, making them of particular interest in the treatment of cardiac disease because they represent a well-characterized and ample source of progenitor cells [67–70]. In vivo demonstration of the same was given by Orlic et al. [71] by direct injection of Lin^−^ c-kit^+^ cells into the infarct region. Number of landmark studies followed then which showed significant improvement in cardiac function when these bone marrow-derived cells were implanted directly or mobilized from endogenous reservoirs. Some actually demonstrated regeneration of contracting cardiomyocytes and improved ventricular function [72–74], while others found beneficial effect independent of tissue regeneration [75–77]. Nevertheless, the improvements seen in ventricular function prompted a number of clinical trials using autologous BMSCs to treat heart failure patients or patients who had suffered an MI.

### 2.7. Mesenchymal Stem Cells (MSCs)

MSCs represent a rare population of cells with absence of HSC markers CD34 and CD133. They are about 0.01% of the mononuclear cell fraction of the bone marrow and are also present in adipose tissue. They are less immunogenic due to lack of MHC-II and B-7 costimulatory molecule expression thereby preventing T-cell responses. They can differentiate into osteoblasts, chondrocytes, and adipocytes [78, 79]. Differentiation of MSCs to cardiomyocytes-like cells was observed under specific culture conditions wherein MSCs were induced to transdifferentiate into cardiomyocyte by 5-azacytidine, a DNA methylation agent [80]. Animal studies have also shown that MSCs have potential for site-specific differentiation into heart muscle cells, vascular-like structures, as well as gap junction protein [80–90]. These results suggest that MSCs act by regenerating functionally effective, integrated cardiomyocytes and possibly new blood vessels. MSCs also have been injected into infarcted myocardium via a catheter-based approach in pigs, resulting in regeneration of myocardium, reduced infarct size, and improved regional and global cardiac contractile function. Importantly, the latter study used allogenic MSCs, which did not produce evidence of rejection [89]. Because MSCs clones can be expanded in vitro and reportedly have a low immunogenicity, they might be used in an allogenic setting in the future as cost-effective “off-the-shelf” allogenic cell product [91]. 

MSCs were derived from adipose tissue; adipose tissue-derived stem cells (ASCs) were first identified by Zuk et al. [92] as a source of adult MSCs. After lineage-specific stimulation, ASCs show multiple lineage differentiation potential. They can differentiate into adipogenic, chondrogenic, myogenic, cardiomyogenic, osteogenic, endothelial, and neurogenic lineages [93, 94]. Adipose tissue is an abundant expandable and easily accessible source of MSCs also evaluated for their therapeutic potential in regenerating heart in animal model after MI [95]. In culture ASCs express cell surface markers similar to those expressed by bone marrow MSCs including CD117 (stem cell factor R), CD29 (beta integrin), CD105 (multilineage differentiation markers), CD54 (intercellular adhesion molecule-1 (ICAM-1), and CD44 [78, 96].

### 2.8. Endothelial Progenitor Cells (EPCs)

Cells with phenotypic and functional characteristics similar to the fetal angioblast also are present in adult human bone marrow [11]. Endothelial progenitor cells (EPCs) represent a subset of HSCs that are able to acquire an endothelial phenotype. In vitro [97–100] EPCs express the HSC markers CD34 and the endothelial marker Flk-1 (vascular endothelial growth factor receptor-2 (VEGFR-2)) [99]. EPC can be isolated directly from the bone marrow or from the peripheral circulation and expanded in vitro. Preclinical trials indicated that EPCs contribute to 1–25% of vessel formation after ischemic injury for several diseases [101]. They promote neovascularization by secreting proangiogenic growth factors and stimulate reendothelialization thereby contribute to vascular homeostasis and perhaps myogenesis [102]. In the animal experiments injection of EPCs into infarcted myocardium improved LVF and inhibited fibrosis [11, 103, 104]. Although there was no change in the noninfarcted regions of the heart, there was a significant reduction in collagen deposition and apoptosis of cardiomyocytes and an improvement in cardiac function on echocardiography [11]. It appeared that neovascularization induced by these cells led to the prevention of apoptosis and LV remodeling and led to some degree of cardiomyocyte regeneration [105]. 

The cell surface antigen CD133^+^ is expressed on early HSCs and EPCs and less than 1% of nucleated BMSCs, and, because these cells cannot be expanded ex vivo, only limited numbers of CD133^+^ cells can be obtained for therapeutic purposes [106].

### 2.9. Fetal and Umbilical Cord Blood Cells (UCBCs)

Because of their prenatal origin, fetal and UCBCs may possess greater plasticity than adult cells. Human umbilical cord blood contains a number of progenitor cell populations, including HSCs and MSCs, in addition to a population of unrestricted somatic stem cells, which have been shown to have proliferative potential [37, 107]. However, animal studies have shown conflicting results with regard to improvements in LVF.

Ma et al. [108] injected human mononuclear UCBCs, a small fraction (*≈*1%) of which were CD34^+^, intravenously 1 day after MI in NOD/scid mice. The cells homed to the infarcted hearts, reduced infarct size, and enhanced neovascularization with capillary endothelial cells of both human and mouse origin. Interestingly, they found no evidence of myocytes of human origin, arguing against cardiomyogenic differentiation. In a rat model of MI [109] UCBCs CD34^+^ improved cardiac function when injected into the peri-infarct rim immediately after MI compared with control animals that received injection of medium. Apart from these, Kögler and colleagues [110] have described a population of cells from human UCBCs called unrestricted somatic stem cells. These cells which are fibroblast like in appearance and adhere to culture dishes are negative for c-kit, CD34, and CD45 and are capable of differentiating, both in vitro and in vivo, into a variety of tissues, including cardiomyocytes. These stem cells [111], when delivered by direct injection at thoracotomy in immunosuppressed pigs after MI, improved perfusion and wall motion, reduced infarct scar size, and enhanced global cardiac function.

## 3. Human Clinical Trials

As already mentioned in the beginning, modern reperfusion strategies and advances in pharmacological management that resolve the ischemia but not the infarct zone have resulted in an increasing proportion of AMI survivors at heightened risk of developing LV remodeling and heart failure. None of our current therapies address the underlying cause of the remodeling process, that is, the damage of cardiomyocytes and the vasculature in the infarcted area. 

BMSCs gained attention as early as in the year 1968, with the first report of their clinical use for restoring the blood and the immune system in children with congenital immunodeficiencies [112]. However, host HSCs, used for blood borne malignancies replace the donor HSCs and they do not have to differentiate into another cell type. Therefore, the revolutionary paper of Orlic et al. [71] with a very provocative finding which suggested that directly injecting HSCs resulted in extensive myocardial regeneration and subsequent various similar reports of animal experiments (Table 3) gave a hope of using stem cells as tool in the hands of mankind for regenerating myocardium [8, 11, 71, 72, 90]. Although their findings were subsequently challenged by Balsam et al. [75], Murry and colleagues [76], and Chien [113], the journey of stem cells as therapy in regenerating the human myocardium had already begun with yet another path breaking clinical study by Strauer et al. (2002) who reported not only improved LVF in human trial but also safety and efficacy of infusing bone marrow mononuclear fraction (BMMNCs) through intracoronary route although in a very small study population [114]. 

Since then, there have been many published studies [115] with different types of cells including composite of BMMNCs, EPCs, MSCs, adipose cells, and cord blood cells. The evidence that precursors of both cardiomyocytes and endothelial cells exist within the mononuclear cell fraction of adult bone marrow forms the basis for the use of BMMNCs in most of the clinical trials to date. After bone marrow aspiration from large bones, most commonly the iliac crest then mixed with heparin; the mononuclear cell fraction is obtained by density gradient centrifugation or sedimentation protocol. The mononuclear fraction is injected into the heart without further ex vivo expansion. In addition, there has been great variability in the number of cells transplanted (1–400 million), and the route of administration has included intracoronary (by using the stop-flow balloon catheter approach), intravenous, epicardial, and intramural methods [116]. The results of clinical trials published to date aiming at progenitor cell-based myocardial repair in patients with AMI are summarized in Table 4(a).

One important point that has to be kept in mind regarding the human trials is that the clinical studies differed significantly from the animal studies: (1) in the animals the infarct-related artery was never reperfused, but cells were directly injected into myocardium in the AMI condition, (2) the majority of these trials utilized relatively unpurified populations of BMMNCs which represent less than 0.1% of stem cells, and none of these trials utilized the Lin^−^c-kit^+^ cells described in animal experiments, and (3) most importantly, the infarction was created in animal by coronary ligation and was not thrombus related. 

The initial pilot studies by Strauer et al. [114], the TOPCARE-AMI [117, 118], the BOOST-trial [119], and the study performed by Fernandez-Aviles [120] as well ours [121] reported nearly identical results—an improvement in global LVEF by an absolute 6 to 9% and reduced LV end-systolic volume (LVESV) at 6 months after cell transplantation. Overall, the published studies demonstrated that the intracoronary infusion of autologous BMSCs is safe and feasible in patients with AMI and on top of the benefits associated with established interventional and medical strategies to promote functional recovery after AMI. Further improvement of LVEF was mostly due to the improved regional wall motion in the infarct border zone. However, there are contradictory reports as well. Janssens and colleagues [122] did not find any improvement in their primary end point after intracoronary transfer of BMMNCs, However, they demonstrated a significant reduction in scar size and an improvement in regional function, but there was no improvement in LVEF (*P* = .36). Their patient population differed from the BOOST trial in that they were reperfused earlier and may therefore have gained only a small benefit from cell therapy because they derived maximal benefit from earlier reperfusion. The beneficial effects observed in most of the pilot phase I/II studies were confirmed in the so far largest double-blind, randomized, multicenter REPAIR-AMI trial [123] which demonstrated not only improved LVF but also showed a reduction in the combined clinical endpoint of death, MI, or revascularization in the BMSCs-treated patients compared with placebo after 1 year followup. Patients with a lower baseline EF (≤48.9%) showed a significant 3-fold higher recovery in global LVEF as well as on clinical end points indicating that patients with more severe MI profit most from BMSCs therapy. Only one larger study, the ASTAMI trial [124], did not show any benefit on LVF parameters. The reason for the failure is considered to be due to their different cell isolation and storage protocol, which significantly affected the functional capacity of the cells. So far, no trial has demonstrated a significant effect of BMSCs transfer on LV end-diastolic volumes (LVEDV), suggesting that unselected BMSCs may have a limited impact on LV remodeling after AMI. Again, larger studies are required to settle this issue. Followup data from the BOOST trial [125] as well as ours [126] show that the improvement of LVEF is maintained after 18 and 24 months, respectively, indicating that BMSCs transfer prevents progression of diastolic dysfunction after AMI.

The therapeutic effects of MSC transplantation after AMI have been investigated in two clinical trials. Chen et al. [127] infused autologous MSCs by intracoronary route and demonstrated no arrhythmias or other side effects. After six months of MSC transfer, regional wall motion and global LVEF were improved, and LVEDV was decreased compared with a randomized control group that had received an intracoronary infusion of saline [127]. Unfortunately, it was not reported whether intracoronary MSC delivery promoted ischemic damage to the myocardium, a complication that had occurred after intracoronary MSC infusions in dogs [128]. Another study by Hare et al. [129] also demonstrated that intravenous allogenic MSCs were safe in patients after AMI with increased LVEF and reverse remodeling. Currently, several studies have been undertaken for allogenic MSCs in clinical trials for myocardial regeneration in the United States under the sponsorship of Osiris Therapeutics. Such an off-the-shelf strategy for cell therapy would potentially make the procedural logistics easier. Taken together, these studies suggested that BMSCs or their selected cell populations are safe and may improve cardiac function by a substantial and clinically meaningful degree following MI. An extensive meta-analysis by Abdel-Latif et al. [130] on eighteen eligible studies (*N* = 999 patients) involving adult BMSCs such as BMNNC, MSCs, and EPCs measuring the same outcomes demonstrated that, as compared to controls, bone marrow transplantation improved LVEF (pooled difference of 3.66%; 95% confidence interval (CI), 1.93% to 5.4%, *P* < .001), reduced infarct scar size (−5.49%; 95% CI: −9.1% to −1.8%; *P* = .003), and reduced LVESV (−4.8% mL; 95% CI: −8.2 to −1.41 mL; *P* = .006). The available evidence suggests that BMC transplantation is associated with modest improvements in physiologic and anatomic parameters in patients with both acute MI and chronic IHD, above and beyond conventional therapy. This further suggests carrying out multicentric randomized large trials targeted to address the impact of intracoronary cell therapy on important outcomes and long-term event-free survival as compared to the conventional therapy.

Studies like those by Werner et al. [131] have also provided evidence of increased survival following AMI in patients with greater number of circulating EPCs. This and positive results of preclinical trials led to human trials to assess safety and feasibility of EPCs [132–134]. The results of these trials showed trend towards improvement of LVF in both acute and chronic ischemia, without adverse effects [133, 135–137].

The ability of injured myocardium to recruit extra-cardiac stem cells following injury is critical in myocardial repair and regeneration. Little is known with regard to the regulatory mechanisms that control the homing and holding of stem cells to injured tissues. The precise time course, kinetics, and factors stimulating bone marrow mobilization remain the subject of intense investigation. Several crucial factors have been shown to promote the mobilization of BMSCs into peripheral circulation, including granulocyte colony-stimulating factor (G-CSF), granulocyte/macrophage colony-stimulating factor (GM-CSF), stem cell factor (SCF), vascular endothelial growth factor (VEGF), hepatocyte growth factor (HGF), and erythropoietin (EPO) [138]. Myocardial ischemia is known to induce several “mobilizing cytokines”, including, but not limited to, G-CSF [139–141], SCF [139–141], VEGF [141–145], stromal derived factor (SDF-1) [139, 141, 145, 146], and EPO [147, 148]. These cytokines may be responsible for the observed homing of BMSCs following MI. Mobilization of BMSCs through cytokine stimulants increases their concentration in the peripheral circulation substantially. In addition to well-recognized HSCs mobilizing agents such as G-CSF and SCF, VEGF, and EPO and statins have been shown to promote EPC recruitment [148–151]. Several clinical trials (Table 4(b)) were carried out with mobilization of BMSCs with G-CSF [152–155]. Abdel-Latif et al. [156] also carried out meta-analysis of clinical trials wherein BMSCs were mobilized with G-CSF. The analysis revealed that G-CSF therapy in unselected patients with AMI appeared safe but did not provide benefit. Subgroup analyses suggest that G-CSF therapy may be salutary in AMI patients with severe LV dysfunction and when started early. Larger randomized studies may be conducted to evaluate the potential benefits of early G-CSF therapy in AMI patients with LV dysfunction.

### 3.1. Safety and Long-Term Benefit of Cell Therapy

Stem cell potency is a double-aged sword, and therefore, although the initial experimental studies confirmed that the infusion of BMSCs do not cause major side effects, several potential issues were raised such as electrical stability, increased restenosis, or progression of atherosclerotic disease. However, none of the clinical studies with BMSCs so far have reported an increased incidence of arrhythmias (as have been seen in some of the myoblast trials), bleeding complications, additional ischemic injury, or promoted inflammatory reaction as no further increase in CRP, and troponin was observed including in our study. 

Restenosis, which was considered as potential side effect by progenitor cell-mediated plaque angiogenesis or plaque inflammation, was only increased using CD133^+^ cells [157, 158]. This is surprising, because the isolation of selected progenitor cells excluding contaminating proinflammatory cells would have been assumed to reduce rather than increase the risk of restenosis and atherosclerotic disease progression. Because CD133^+^ cells were isolated by using a mouse antibody, one may speculate that the remaining antibody might have elicited a local proinflammatory reaction despite the failure to detect systemic antimouse antibodies in the patients. All other studies did not observe an augmented risk for restenosis [159]; if anything, there was a decreased necessity for revascularization procedures in the REPAIR-AMI trial [123]. 

Intramyocardial calcification which was reported to occur in murine models of MI after direct injection of unpurified BMSCs or MSCs [160, 161] was not reported in the various clinical trials as reported by MRI imaging. This may be explained by the enrichment of mononuclear cells by density gradient centrifugation used in the majority of the clinical studies. 

It had been discussed that the proangiogenic capacity particularly of EPCs might relate to an increased tumor vascularization. However, during followup of the available studies, no increased incidence of cancer was seen in BMSCs-treated patient. Most of the clinical trials did exclude patients with known tumors. It is unclear whether a single application of EPCs is sufficient to promote tumor growth. However, because of the low incidence of such events, this needs to be carefully monitored in the future. 

An important issue is whether the improvement seen during the initial 6 months after cell therapy is maintained for a prolonged time. Careful evaluation of the 18 months followup data of the BOOST trial indicates that the EF of the cell therapy group is maintained from 6 to 18 months followup [125]; however, the difference between the cell therapy and the control group was no longer statistically significant. The small number of patients (30 per group) may preclude detecting a statistical difference between the 2 groups. The long-term 5-year followup MRI-derived data of the TOPCARE-AMI trial showed that the EF was maintained and even further augmented in the treated patients, in parallel with a sustained reduction in NT-proBNP serum levels suggesting a sustained beneficial effect on long-term LV remodeling (S. Dimmeler and A. M. Zeiher, unpublished data). In our study also the improvement seen at 6 months in LVF was sustained at 24 months. However, longer-term followup in larger-scale randomized trials will finally address this important question. Overall, the clinical data available at present indicate that cell therapy with bone marrow-derived cells is feasible and safe at least for the duration of followup presently available (up to 5 years for the initial studies).

## 4. Mechanism of Myocardial Repair

One could see that, although the early phase of research in cardiac repair aimed at histologic outcomes, the human trial of last five years demonstrated improvement of heart's function as their clinical end point and have erroneously reasoned that, because ventricular function was improved, the heart was regenerated. This shift towards physiology made mechanism less evident. There is still controversy as to whether actual differentiation occurs versus large cell fusion with resident myocytes. This is because on one hand the myocyte deficit in infarction-induced heart failure is on the order of one billion cardiomyocytes and on the other hand the documentation of LVF improvement within 72 hours is far earlier than would be expected for cell regeneration of any meaningful extent [162]. 

**Figure 1:**
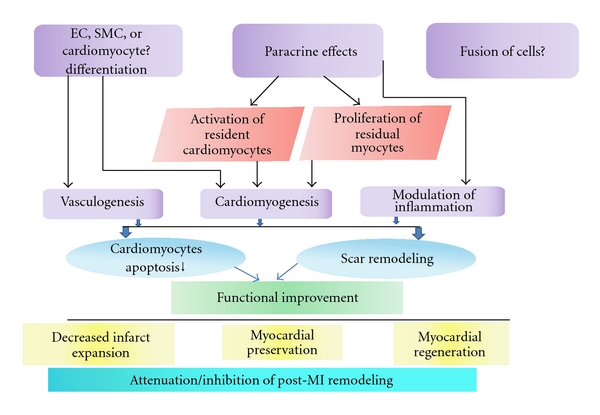
Mechanism of action of stem cells for cardiac functional improvement.

The fact that after transplantation of hundreds of millions of cells, less than 2% of the cells are actually still present in the tissue within 2 weeks of implant; the prevailing concept of stem cell efficacy has shifted toward the cytokine-paracrine hypothesis [163]. It has also been proposed that through paracrine mechanisms stem cells release angiogenic ligands, protect cardiomyocytes from apoptotic cell death, induce proliferation of endogenous cardiomyocytes, and may recruit resident CSC (Figure 1). Indeed, various studies showed that progenitor cells secrete survival factors such as endothelial growth factor, stromal-derived factor (SDF-1), angiopoietin 1, hepatocyte growth factor, insulin-like growth factor 1, and periostin [77, 164–169], thymosin b4 which promotes wound healing or the Wnt antagonist secreted frizzled-related protein 2 (SFRP-2) which protects cardiomyocytes from hypoxia-induced apoptosis [170–172] and thus stimulate tissue recovery after ischemic injury and minimize the infarct size [165, 167, 173–176]. Regardless of the mechanisms, there appears to be general agreement that stem cell therapy has the potential to improve perfusion and contractile performance of the injured heart.

## 5. Issues to Be Addressed in the Future Studies

The ultimate aim of the cellular transplantation remains to be the regeneration of lost heart muscle along with the reversal of the remodeling process. It is possible that the apparently variable results among different trials are secondary to differences in the protocols. Despite growing clinical experience, use of these heterogeneous parameters along with various clinical end points among human trials have left us with fundamental questions regarding the use of the ideal cell type; the number of cells needed to be delivered for maximal efficacy; optimal isolation, purification, and storage techniques; ideal route of delivery; ideal time of administration after injury to improve the efficacy of this therapy as well as for this therapy to be included under treatment guidelines. The trials discussed above were not powered to address the effects on these hard clinical end points but can give us some direction towards standardization of the therapy on above parameters. 

### 5.1. Which Cell Populations Should Be Delivered?

While the ideal cell type for stem cell therapies remains to be determined to date, bone marrow-derived stem cells, isolated from whole bone marrow aspirate, remain the most commonly used cell type for human studies. Unfractionated bone marrow cells gained advantage over above cells due to many reasons. It has the feasibility of procuring, no requirement of in vitro expansion and above all the availability of mixed population of cells with characteristic for differentiating into various populations of cells. And of course it has no ethical issues. However, importantly MSCs have also emerged as most promising cell population with their inherent property of transdifferentiating into cardiomyocytes and to be tolerated by the immune system giving us the most convenient “off-the-shelf” reagent.

### 5.2. What Number of Cells Should Be Given?

Murry et al. [162] have pointed out that number of cells administered reported studies range by as much as 6700-fold. Myocardium contains approximately 20 million cardiomyocytes per gm of tissue [177]. The average left ventricle approximately weighs 200g and therefore contains approximately 4 billion cardiomyocytes. To cause a heart failure, an infarct needs to kill approximately 25% of the ventricle (for comparison, infarcting 40% of the ventricle results in acute cardiogenic shock) [178]. Therefore, the myocyte deficit in infarction-induced heart failure is in the order of one billion cardiomyocytes. True cardiac regeneration would therefore require restoring approximately one billion cardiomyocytes and ensuring their synchronous contraction via electromechanical junctions with host myocardium.

### 5.3. When Dose Cells Should Be Transplanted?

In the first 48 hours of AMI attack, debridement and formation of a fibrin-based provisional matrix predominates before a healing phase ensues [179]. At the initial 3-4 days after MI cell adhesion, molecule concentration which has not yet declined may promote the transplanted cells into inflammatory process than in the formation of functional myocardium [180]. It is only by 7th day after MI that VEGF concentration peaks and cell adhesion molecule concentration declines [181, 182]. By 2 weeks after scar formation, the benefits achieved due to cell transplantation are reduced. Therefore, the ideal time point of transplantation remains 7–14 days [105]. This was very much evident in the REPAIR AMI trial [123] wherein patients being treated up to 4 days after the MI showed no benefit, whereas later treatment (day 4 to 8) provided an enhanced improvement of EF during follow-up. This suggests that microenvironment after AMI changes during the first week after reperfusion, thereby modulating the homing or the subsequent functional activity of the infused cells and cell homing might be best after a few days rather than immediately after reperfusion. Further studies are warranted to prospectively address this question [183].

### 5.4. Cell Processing

Standardization of cell isolation protocols which have a major impact on the functional activity of bone marrow-derived progenitor cells is also a crucial issue. The comparison of the cell processing of ASTAMI and REPAIR trials highlighted a very crucial point that the assessment of cell number and viability may not entirely reflect the functional capacity of cells in vivo [184]. Additional functional testing appears to be mandatory to assure proper cell function before embarking on clinical cell therapy trials. The recovery of total cell number, colony-forming units (CFU), the number of MSCs, and the capacity of the isolated BMSCs to migrate in response to SDF-1 was significantly reduced when using the ASTAMI [124] protocol of Lymphoprep, storage in NaCl plus plasma compared with the REPAIR [123] protocol of Ficoll, and storage in X-vivo 10 medium plus serum. Comparison of the individual steps identified the use of NaCl and plasma for cell storage as major factors for functional impairment of the BMSCs in ASTAMI trail.

### 5.5. Specific Setup Required for Clinical Cell Therapy to Ensure Quality Control and Safety

The application of BM-derived cells to patients is a complicated process starting from the operating theatre, then moving to the laboratory, and finally returning to the operating theatre or cardiac catheterization laboratory. Aseptic techniques are used throughout to control contamination. In today's clinical practice such a process must comply with good manufacturing practice (GMP) to reduce risks to the eventual recipients. Accordingly, cell therapy centers should be GMP accredited, wherein trained personnel operates under specific facilities (air filtration systems, clean room, and cell culture laboratory facilities) following the GMP standards. Similar setups for hematopoietic stem cell therapy should be adopted in hospitals worldwide. Cell processing may inadvertently expose human cells with a variety of hazards and contaminations. The major concern is zoonotic contamination, as animal-derived materials (e.g., fetal calf or bovine serum) are commonly used in cell cultures. This problem could be overcome by using commercially available serum-free media (with growth supplements). In the meantime, Autologous Human Serum may be considered as a replacement to animal serum. Finally, prior to the delivery in patients, cell supernatants must be tested for infectious agents at different time points dependent on the specific protocol [183].

### 5.6. Which Application Method Is the Most Efficient?

The most commonly utilized method of stem cell delivery is the transvascular approach. It is suited for the treatment of recently infarcted and reperfused myocardium which delivers maximum cells to the site of injury. Cells are delivered through the central lumen of an over-the-wire balloon catheter during transient balloon inflations to maximize the contact time of the cells with the microcirculation of the infarct-related artery. This stop-flow technique is relatively easy to perform within less than an hour and enhances cell retention within the infarcted area. Direct needle injection of the stem cells into the infarcted regions of the heart that requires an open-chest procedure may not be possible all the time for human patients [106]. 

The use of IV therapy could be more effective considering that the cells have the advantage of reaching the tissue and vessels surrounding the infarct region. When using direct infusion, the cells primarily reach the area that they are injected into, whereas IV administration is not necessarily limited to the immediate infarcted region. It is possible that the stem cells will also repair areas of the heart damaged during any previous injury and not detected by imaging, therefore preventing any future problems in that region. Since IV administration is safer for use on humans than catheterization, clinical trials utilizing IV therapy with humans would be a critical step for the standardization of stem cell treatment. Hare et al. [129] had used intravenous approach to deliver the allogenic MSCs to the infarct region with positive outcome.

### 5.7. Development of Safe and Effective Cell Tracking Modalities

Because the mechanism through which cell therapy acts is still being characterized, clinical trials that establish mechanistic correlates will be most helpful. For example, studies using MRI in patients suggest that cell therapy might alter the rate of infarct repair or influence the amount of scar contraction [122]. Positron emission tomography studies have demonstrated glucose uptake and enhanced myocardial blood flow in cell-engrafted regions [117, 119, 128, 185, 186] which provide important information regarding effects on tissue metabolism and perfusion. Another very useful mechanistic end point for clinical trial is the ability to track the cells after they are implanted, for instance, through use of paramagnetic particles visible by MRI [187], positron emitting isotopes [188], or molecular tracers [189]. Finally tissue-based analyses should be included in clinical trial design, either by evaluation of explanted hearts at the time of the transplantation [190] or by autopsy of patients who die following cell therapy [191].

The huge scope of the problem from the bench to the bedside and back again led to the establishment of consortia. The Cardiovascular Cell Therapy Research Network, which comprises 5 institutions and is sponsored by the National Heart, Lung, and Blood Institute, will address a specific series of questions over a 5-year period. Broadly speaking, the major objectives are to develop phase 1 and 2 clinical trials for cell delivery for left ventricular dysfunction (AMI and chronic heart failure) while defining parameters and models for successful translation of newer cell types [192].

## 6. Cell-Enhancement Strategies

Apart from the standardized steps obtained from the clinical trials to continue the research at bedside, there needs to be done a lot at bench side to make this trial effective and more specific. Pretreatment of the targettissuesenhance active cell recruitment, survival, and retention; the potential strategies include modification of the target region by low-energy shock waves or ultrasound-mediated destruction of microtubules in the coronary circulation that increase retention of EPCs, BMMNCs, and MNCs. 

Many cytokine and paracrine factors that favorably affect angiogenesis, inflammation, cytoprotection, metabolic modulation, and apoptosis have been identified. Activation or introduction of chemotactic factors such as SDF-1 or its receptor CXCRF4, high-mobility group box-1 (HMGB-1), *β*2 integrin, and endothelial nitric oxide synthase to attract cells to the damaged area can be carried out. Another area of interest relates to the decline in the number and functionality of autologous stem cells in association with comorbid conditions and whether such “sick” cells can be functionally rejuvenated before transplant. Experimental studies of cell-enhancement strategies have identified a number of novel and intriguing options for improving survival, retention, integration, and homing. These include through genetic modification of stem cells before transplant [193, 194], that is, the transduction of cells with prosurvival genes (e.g., the protein kinase Akt, telomerase reverse transcriptase (the active subunit of telomerase), vascular endothelial growth factor, and integrin-linked kinase) and the pretreatment of cells with small molecules (e.g., statins, P38 inhibitors, and endothelial nitric oxide synthase (NOS3)), so as to activate the Akt/NOS3 pathway [195–197].

**Finally, next directions of cardiac cell therapy include following [197]:**

(1) The study of the array of bioactive molecules that are secreted by stem cells, which have been demonstrated to induce neovascularization, modulate inflammation, fibrogenesis, cardiac metabolism, and contractility, increase cardiomyocyte proliferation, and activate resident stem cells. The exhaustive analysis of this “secretomes” of BMMNCs, MSCs, or EPCs would lead to a better understating of the mechanisms of action of the cells and to a hypothetical protein-based therapy (off-the-shelf, noninvasive, systemic, and repetitive administration); (2) the use of different sources of pluripotent stem cells, like ESCs, spermatogonial stem cells, and oocytes. A new era has been initiated with the possibility of reprogramming adult cells (skin fibroblasts) to a pluripotent state by retroviral transduction [26, 198]. These iPSs show the characteristics of ESCs and can differentiate to cardiomyocytes. New retroviral vectors and even nonviral vectors have been developed to reduce the risk of mutagenesis, and genetic modification of cells with suicide genes have been proposed to reduce the risk of tumor formation; (3) the creation of bioartificial hearts after a process of decellularization with detergents, abstention of the underlying extracellular matrix (cardiac architecture), and stem cell repopulation [199]. The “acellular” heart can then be reseeded with CSCs or EPCs, showing contractile activity in animal models. This new approach of tissue bioengineering has opened a fascinating era in cardiovascular medicine.

### 6.1. Strategies for Generating Cardiac Myocytes

Pretransplantation conditioning/specification of cells to the cardiac phenotype has been studied

#### 6.1.1. ESCs and iPS

The cardiac differentiation potential of iPS was carried out by selecting the mesodermal marker Flk-1 (VEGFR-2) from differentiating iPS and then a further coculture with OP9 cells. It resulted in the induction of cardiac myocytes [200]. These myocytes have also been demonstrated to be functionally and structurally similar to those derived from ESCs, but the yield was still low.

Pretransplantation conditioning/specification of cells to the cardiac phenotype has been widely explored in studies with ESCs. The induction of spontaneous beating in vitro of ESC cultured as embryoid bodies with the addition of members of the transforming growth factor family proteins (TGF*β*1, BMPs) appears to be a common approach. Coordinated beating areas in the cultures are then isolated, characterized for the expression of cardiac genes, and used for transplantation [15, 19, 201].

A noticeable work was performed by Kehat et al. [202], where the electrophysiology coupling of human ESC-derived cardiac myocytes (ESC-CM) was completely dissected with in vitro cocultures of these cells with rat postnatal cardiac myocytes and in vivo after transplantation in animal models of cardiac dysfunction.

Laflamme et al. [203] took a two-tiered approach to ESC-based therapy of AMI by preconditioning the cells initially toward cardiac lineage and then treating the cells with a prosurvival cocktail 3. The directed differentiation of hESCs by treatment with BMP4 and Activin increased the yield of myocytes to 30%. The prosurvival cocktail markedly increased cell engraftment and survival. This combination of treatments greatly augmented remuscularization and cardiac function.

#### 6.1.2. Adult Stem Cells

Adult stem cells pretransplantation specification to the cardiac phenotype has received some attention, although not in the clinical realm.

MSCs have been shown to express basal levels of major cardiac proteins such as cardiac myosin, actinin, and others, have capacity to readily occupy the infarcted tissue before cardiac fibroblast do, avoiding fibrotic scar formation, as well as secrete survival factors that in turn maintain remaining cardiac myocytes viability and induce endogenous cardiac stem cells to differentiate and recover lost tissue/function. However, cardiac differentiation of MSCs integrated into the infarcted cardiac tissue, in terms of sarcomeric organization and coordinated beating has not been reported to date. In a recent publication pretreatment of MSCs with a combination of growth factors, basic fibroblast growth factor; insulin growth factor-1, and bone morphogenic protein-2 (FGF2/IGF-1/BMP2, resp.) induced cardiac protein expression and antiapoptotic signals in adjacent cardiac myocytes in vitro. This study detailed the importance of gap junction proteins such as conexin-43, which has enhanced expression in hearts transplanted with growth factor pretreated MSC [204].

Enhanced expression of cardiac proteins such as Troponin-I and conexin-43 after 7 days of treatment with TGF*β*1 was observed in immunogenic selected MSCs expressing the oncogene c-kit also known as CD117. Li et al. demonstrated that CD117^+^ cells transplanted into infarcted hearts improved cardiac function determined by cardiac wall thickness and shortening fraction [205].

## 7. Philosophical and Ethical Issues

Because of the innovative nature of these treatments, differences between animal and human physiology, limited experience with these cells in humans, and the high hopes of desperate patients for whom no alternative effective treatment currently exists, the focus should be on reducing risks and providing rigorous evidence of efficacy and safety within the ethical guidelines. In general, the fundamental regulatory and ethical requirements that are used in drug and other clinical trials apply equally to cell therapy [206, 207]. In addition, the use of embryonic material for research will require formulation of additional safeguards by which cells are manipulated and engineered, which in turn will be influenced by attitudes of governmental administrations [208]. The lack of precedent in this area of science mandates a close interaction among regulators, scientists, clinicians, and the public because the potential for misunderstanding on all sides is considerable. Other issues include those of ownership of cell lines, intellectual property, patents, collection of blood in minors (i.e., umbilical cord blood donations), the potential effect of conflict of interest on research study recruitment, and analysis of results [209–211]. Because use of patient's autologous stem cells does not directly involve intellectual property, funding of stem cell research by the biotechnology industry has been limited to either cell isolation or delivery devices. Perhaps this situation will change with the emergence of new stem cell lines that are not autologous and the development of unique processing capabilities, but these issues in turn have raised concerns with regard to ownership, price control, and the availability of cell lines. For the present, expensive randomized controlled clinical trials are funded primarily by nonindustrial sources, introducing a difficult challenge for the academic community of disease [208].

Some specific ethical recommendations have been given for Randomized Clinical Trial (RCT) with stem cells [210]. They include the following.

1. Phase I-II trials should enroll participants in late stages of serious illness, such as persons with advanced or refractory disease, but not so ill that they are at greatly increased risk for adverse events.
2. Use a proper control group, in order to evaluate the positive effects of treatment and to ascribe culpability to any MACE seen with cell therapy. Then stem cells can be offered to the control group at the conclusion of the trial if the results show short-term benefit (cross-over).
3. Use clinically meaningful endpoints.
4. Coordinate scientific and ethical review, judging the potential clinical benefit of the treatment and assessing the scientific justification for the trial, including proof-of-principle and preclinical data on safety and dosage.
5. Verify that participants clearly understand the features of the trial. Since a comprehensive informed consent form may not prevent misconceptions about the trial, additional information should be given to those patients with significant misunderstandings. Participants should appreciate that researchers may not know whether or not the stem cell treatment will be beneficial, that animal studies might not predict effects of the cells in humans, and that unexpected adverse events may occur.
6. Ensure publication of results, even negative ones. For the interest of patients, researchers, and sponsors, negative findings cannot be withheld from publication.

The Task Force of the European Society of Cardiology on stem cells and repair of the heart was created in 2006 to investigate and regulate the role of progenitor/stem cell therapy in the treatment of cardiovascular disease. It was almost four years ago that this group of experts and opinion leaders stated the type of studies needed [212]. On the other hand, the Task Force stated that small uncontrolled trials with BMMNCs should be avoided, as they are unlikely to add anything new to the field. 

The points stated were the following:

1. further large, double-blind, multicentric controlled RCT for the use of autologous BMSCs in the treatment of AMI. The patient population should be all those presenting within 12 hours of AMI and treated with immediate revascularization, be it primary angioplasty or fibrinolysis;
2. double-blind, controlled RCT for the use of autologous BMSCs in the treatment of AMI in those patients presenting late (>12 hours) or who fail to respond to therapy (candidates for “rescue” angioplasty). Although these groups may represent a small proportion of all patients with AMI, their prognosis remains poor;
3. double-blind, controlled RCT for the use of autologous BMSCs or SM in the treatment of ischemic heart failure. At some stage, the role of autologous stem/progenitor cells in the treatment of cardiomyopathies (in particular, dilated cardiomyopathy) will need to be examined;
4. a series of well-designed small studies to address safety or mechanism to test specific hypotheses (i.e., studies with labeled cells or to investigate paracrine or autocrine mechanisms). Such hypotheses would have arisen from basic science experiments;
5. studies to confirm the risk/benefit ratio of the use of cytokines alone (i.e., G-CSF) or in conjunction with stem/progenitor cell therapy. This Task Force also underlined the necessity for studies with hard clinical endpoints, MACE, subjective benefit, and economic gain;
6. another key point is standardization, both in outcome measures and in the processing of cells (better achieved in specialized centers following Good Manufacturing Procedure routines), in order to derive meaningful comparisons.

## 8. Conclusion

The Orlic trial almost ten years ago that fueled excitement of using stem cells for cardiac regeneration was itself under criticism. However, often “Science Profits from Mishaps.” Many of the bigger discoveries and inventions have followed such traumatic experiences. Had Andreas Gruentzig not embarked on clinical applications after experimenting on just 8 dogs, a new field of interventional cardiology would not have emerged. Therefore, the risk of exposing patients to possible adverse outcome of any new treatment must be weighed against the risk of depriving all patients of new treatment to alleviate sufferings or prolong the life. The argument that these trials should be delayed till mechanisms are further understood will prevent large number of patients from therapeutic approaches that may improve their clinical outcome. There is a wealth of preclinical and early clinical data showing safety, feasibility, and early efficacy of adult cell-based therapy. The apparent lack of immediate commercial or industrial interest should not discourage the scientific community from adopting a disciplined strategy in pursuing this field. Now only clinical trials can lead to optimization of it. Adult stem cell therapies should therefore precede randomized, placebo-controlled double-blind clinical trials. Another compelling argument for initiating clinical research is that the results of these investigations often provide pivotal insights that allow a new field to advance. For now, the main challenge is to improve the translation of cellular and molecular concepts into clinically relevant endpoints so that stem cell therapy in conjunction with current treatment modalities may help to further reduce the mortality and improve the quality of life in MI patients.

## Figures and Tables

**Table 1:** Evidence of myocardial regeneration.

Study	Findings
Kajstura et al. 1998 [3]	14 × 106 myocytes in mitosis by confocal microscopy.

Beltrami et al. 2001 [4]	4% myocytes in mitosis by labeling with nuclei antigen Ki-67.

Hierlihy et al. 2002 [5]	Endogenous resident cardiac lineage negative [L-] C-Kit + stem cells differentiated into all three main myocardial cell types; myocardial, endothelial, and smooth muscle cell types.

Laflamme et al. 2002 [6] Quaini et al. 2002 [7]	Sex-mismatched cardiac transplantations, homing of recipient's progenitor cells in the myocardium was demonstrated. In the procedure Y chromosome in situ hybridization was used to track the male cells in the female allografts coupled with immunostaining to define the identity that these cells had acquired.

Jackson et al. 2001 [8] and Bittira et al. 2003 [9]	Marrow-derived progenitor cells circulate and home to injured tissues similarly to leukocytes, where they contribute to the formation of new tissues.

**Table 2:** Major cell types with potentials for cardiac cell therapy.

Type	Markers	Advantages	Disadvantages
*Embryonic stem cells (ESCs) * Blastocysts (inner cell mass)	—	Totipotent and highly expandable	Immunosuppression required, ethical debate, lack of availability, and tumour potential

*IPS (induced pluripotent cell) * Fibroblast (by reprogramming adult somatic cells with genes regulating ESC pluripotency)	—	Pluripotent indistinguishable from ESCs at the epigenetic and functional levels. Embryonic stem cell like autologous adult cells for cell therapy	Tumourigenesis

*Adult/Fetal cardiomyocytes *	Isl^+^, Lin^−^ c-kit^+^ Sca-1^+^ cardiosphere cells, SP cells	Multipotent Cardiomyocyte phenotype Electro-physiologically compatible	Immunosuppression required, ethical debate, short survival, and limited supply

*Skeletal myoblasts satellite cells *	CD56^+^	Autologous transplantation, lack of immunogenicity and high yield and fatigue resistant, slow twitch fibers	Electrophysiologically uncompatible, lack of gap junction, arrhythmogenic

*Hematopoietic stem cells * Bone marrow/peripheral blood	CD34^+^, CD45^+^, CD133^+^	Multipotent, lack of immunogenicity and autologous transplantation, different lineage of cells	Quantum of cell population not adequate

*Mesenchymal Stem Cells * Bone marrow Stromal/muscle, skin, and adipose tissue	Adhesion molecules (ALCAM/CD44) Antigens (SH2/SH3/SH4/STRO-1)	Allogenic/autologous transplantation, lack of immunogenicity (lack MHCII and B7 expression), pluripotent and cryopreservable for future use	Requires expansion

*Endothelial progenitor cells* Bone marrow/peripheral blood	CD133^+^	Autologous transplantation, monopotent, lack of immunogenicity	Need for expansion because of limited supply

**Table 3:** Animal experiments demonstrating myocardial generation with BMSCs.

Study	Findings
Tomita et al. 1999 [90]	Transplantation of autologous bone marrow cells to stimulate angiogenesis in the recipient ischemic myocardium. Functional improvement was observed only in recipients of the mesenchymal stem cells that had been treated with 5-azacytidine.

Orlic et al. 2001 [71]	Haematopoietic stem cells injected were demonstrated to occupy the infarcted region and resulted in extensive myocardial regeneration.

Jackson et al. 2001 [8]	The engrafted SP cells (CD34(−)/low, c-Kit(+), Sca-1(+)) or their progeny migrated into ischemic cardiac muscle and blood vessels, differentiated to cardiomyocytes and endothelial cells, and contributed to the formation of functional tissue.

Kocher et al. 2001 [11]	Systemic infusions of human bone marrow-derived endothelial cell precursors were able to intercept the remodeling process of the left ventricle. The observed neovascularization prevented apoptosis of hypertrophied myocytes reducing collagen deposition and subsequent scar formation. Posttransplantation ventricle function improved as well.

Orlic et al. 2001 [72]	That mobilization of animal's own bone marrow with G-CSF before and after myocardial infarction in mice resulted in growth of new cardiomyocytes in the infarct zone, improved ventricular function, and substantially decreased mortality by 68%.

**Table:** (a) Summary of major cell-based clinical trials

Study	Method of delivery	Patients treated/control	Placebo/control	Cell type cell/number or dose	Time of cell delivery (days after MI)	Results
Strauer et al., 2002 [114] (Germany)	IC	20/10	Case controlled	BM-MNC 9–28 × 10^6^	7	Improved contractility and reduced infarct size at 6 months

TOPCARE-AMI, Assmus et al. 2002 [117], Schächinger 2004 [118] (Germany)	IC	30/29	Control Nonrandomized open-labeled	BM-MNC 2.4 × 10^8^ CPC 1.3 × 10^7^	3 to 7	Improved LVFE and reduced infract size at 4–12 months

BOOST, Wollert et al. 2004 [119],	IC	30/30	Control	BM-MNC 24 × 10^9^	6	Improved EF at 6 months, increased regional contractility,
Meyer et al. 2006 [125] (Germany)						no difference at 18 months

REPAIR-AMI, Schächinger et al. 2006 [123] (Germany)	IC	102/102	Placebo	BM-MNC 2.4 × 10^8^	4	Improved EF and reduced infarct size at 4 months

Fernandez et al. 2004 [120]	IC	20/13	Control	BM-MNC 11–90 × 10^6^	10–15	Significant functional improvement and reduced infarct size

Janssens et al., 2005 [122] (Belgium)	IC	33/34	Placebo	BM-MNC 3.0 × 10^8^ cells	1	Decrease scar size but no improvement in LVEF at 4 months

ASTAMI, lunde et al. 2006 [124] (Norway)	IC	50/50	Control Randomized + placebo controlled	BM-MNC 8.7 × 10^7^	5 to 8	No difference at 6 months

Shah et al. 2007 [121, 126] (India)	IC	20/10	Control Open-label nonrandomized	BM-MNC 13.4 × 10^7^	6 to 8	Improved LV function at 6 months and sustained at 24 months

Chen et al. 2004 [127] (China)	IC	34/35	Placebo Controlled	MCSs 48–68 × 10^10^	18	Inc LVEF, Inc regional contractility, increase viability of infarct zone/wall after 3 and 6 months

Hare et al. 2009 [129] (USA)	IV	39/21	Double-blind placebo controlled	MSCs 0.5, 1.6, 5 million cells/kg	1, 2, 3, 6 months followup	Improved LVEF and reverse modeling

IC: intracoronary, IV: intravenous, BM-MNC: unfractionated bone marrow mononuclear cells, CPC: Circulating progenitor cells, MSCs: mesenchymal stem cells.

**Table:** (b) Summary of major cell-based clinical trials

Study	Method of delivery	Patients treated/controlled	Placebo/control	Cell type, cell number, or dose	Time of cell delivery (days after MI)	Results
Ince et al. 2005 [152] First Line-AMI (Germany)	Mobilization of G-CSF	15/15	Randomized + controlled	BM-MNC CD34+	1–6	After 4 and 12 month followup improved LVEF and systolic wall thickness

Ripa et al. 2006 [153], STEMMI (Denmark)	Mobilization of G-CSF	39/39	Randomized + placebo controlled	BM-MNC CD34+	1–6	After 6 month followup systolic wall thickness ↑ viability of infarct zone/wall
Zohlnhöfer et al. 2006 [154], REVIVAL (Germany)	Mobilization of G-CSF	56/58	Randomized + placebo controlled	BM-MNC CD34+	1–6	After 4 and 6 month followup No effects

Engelman et al. 2006 [155], G-CSF-STEMI (Germany)	Mobilization of G-CSF	22/22	Randomized + placebo controlled	BM-MNC CD34+	1–5	After 4 and 6 month followup No effects
